# Fatigue and quality of sleep jointly influence the association between physical activity and health-related quality of life in patients with chronic kidney disease: a cross-sectional study

**DOI:** 10.3389/fneph.2025.1649578

**Published:** 2025-11-18

**Authors:** Véronique De Gucht, Dion H. A. Woestenburg, Vesna Vrecko Pizzulin, Krister Cromm

**Affiliations:** 1 Research Group of Health, Medical and Neuropsychology, Institute of Psychology, Leiden University, Leiden, Netherlands; 2 Methodology and Statistics Research Unit, Institute of Psychology, Leiden University, Leiden, Netherlands; 3 Division of Surgery, University Medical Centre Ljubliana, Ljubliana, Slovenia; 4 Fresenius Medical Care Deutschland GmbH, Global Medical Office, Bad Homburg, Germany; 5 Department of Psychosomatic Medicine, Center of Internal Medicine and Dermatology, Charité Universitätsmedizin Berlin, Corporate Member of Freie Universität Berlin, Humboldt-Universität zu Berlin, Berlin Institute of Health, Berlin, Germany; 6 Center for Patient Centered Outcomes Research, Charité Universitätsmedizin Berlin (CPCOR), Berlin, Germany

**Keywords:** chronic kidney disease, fatigue, sleep, physical activity, HRQoL, renal transplantation, renal replacement therapy, dialysis

## Abstract

**Introduction:**

Fatigue is a prevalent and burdensome symptom in Chronic Kidney Disease (CKD), with major impact on Health-Related Quality of Life (HRQoL). Physical activity has been linked to improvements in both fatigue and HRQoL. This study examined whether physical activity relates to HRQoL indirectly through fatigue and whether this relationship is moderated by sleep quality.

**Methods:**

A total of 465 CKD patients (mean age = 53.78 years; 50% female) participated in the study. Fatigue, physical activity, HRQoL, and sleep quality were assessed and compared to general population norms and across treatment modalities using t-tests and ANCOVAs. Mediation, moderation, and moderated mediation analyses were conducted.

**Results:**

CKD patients reported lower physical activity levels, HRQoL, and sleep quality, and higher fatigue than the general population (all *p*s <.001). Among treatment groups, transplant recipients showed the most favorable outcomes, while patients without renal replacement therapy reported the poorest. Higher levels of physical activity were associated with better HRQoL indirectly through fatigue, with small to moderate effect sizes. Stronger associations observed in those reporting better sleep quality.

**Discussion:**

These findings indicate that physical activity is associated with better HRQoL in CKD patients through its relationship with fatigue, particularly among those with good sleep quality. Future research should explore fatigue across CKD stages to optimize interventions that target both physical activity and sleep.

## Introduction

Fatigue appears to be the main complaint in chronic kidney disease (CKD). In a healthy population, fatigue is reported in 5% to 45% of the cases (1). The prevalence of (chronic) fatigue in CKD is substantially higher and varies depending on the study from about 50% to 89% in hemodialysis (HD) (2). Very few studies focused however on peritoneal dialysis and transplant populations (3). Given its prevalence and burdensome nature, fatigue has been identified as a highly prioritized symptom to treat and investigate (4).

The biopsychosocial model of fatigue in CKD patients (5) hypothesizes fatigue is precipitated by physiological factors [uremia, anemia, blood pressure, inflammation (2, 6, 7)] and may be perpetuated by psycho-behavioral [psychological distress, sleep disturbances, physical activity, smoking, and cognitions (2)], and social factors (e.g. social support). However, specific determinants and causal mechanisms of fatigue in CKD patients are not well understood due to its complex multifaceted etiology (2, 8, 9) and the lack of longitudinal studies (2).

Fatigue has a profound direct and indirect negative effect on individuals’ functioning and quality of life. Consequences of fatigue are directly reflected in impaired functionality (10), disease progression, increased risk of hospitalization (11), and lower physical and mental Health-Related Quality of Life (HRQoL) (2, 12–14). Indirectly, fatigue may predict mortality through distress, impaired functioning and its consequences (15). It should, however, be noted that both fatigue and HRQOL differ by CKD treatment and modality (16, 17).

Poor sleep quality proved to be associated with more fatigue in patients on maintenance hemodialysis, while prolonged fatigue in these patients was associated with mortality (13). According to a review in hemodialysis patients, fatigue is especially related to poor physical HRQoL (18). Fatigue in patients with CKD, especially in patients on long-term dialysis, also leads to muscle wasting, resulting in inactivity or reduced physical activity which are in turn related to mortality (19, 20).

Experiential evidence shows physical activity decreases fatigue in CKD patients (9) and improves HRQoL (21). It is hypothesized physical activity has a positive, multifaceted effect (9) on fatigue due to its beneficial effect on pathophysiologic mechanisms of fatigue in CKD populations such as improved mental health, a positive effect on the cardiovascular and muscular system and reduced inflammation (9, 22, 23) which are evident across the whole spectrum of CKD (19). In addition, a systematic review by Zhao et al. (24), based on 13 randomized controlled trials, demonstrated that exercise interventions have a positive impact on fatigue as well as on both physical and mental HRQoL in CKD. Therefore, physical activity could be a promising target to alleviate fatigue in this population (9).

Sleep has an important regenerative and restorative function in an individual’s health (25). Unhealthy sleep, characterized as inappropriate length or quality, results in a myriad of negative consequences such as impaired daily functioning, poor quality of life (26), reduced well-being (25), and disease progression (27). Diminished sleep quality is assumed to be a significant determinant of fatigue in CKD patients (13, 28, 29).

Evidence suggests that physical activity benefits both fatigue and HRQoL in patients with CKD, and studies in healthy populations indicate that the association between physical activity and fatigue depends on sleep quality (30, 31). Yet, despite the high prevalence of fatigue and its association with poor sleep in CKD, little research has examined how these factors jointly shape the relationship between physical activity and HRQoL, leaving an important gap in understanding their combined roles.

Based on previous studies in healthy and CKD samples, we hypothesize that physical activity is indirectly associated with better physical and mental HRQoL through lower fatigue, and that this indirect association is stronger among CKD patients reporting better sleep quality.

The current study focuses on the following research questions (RQs):

- RQ-A: Compare fatigue, sleep, physical activity, and physical and mental HRQoL between patients with CKD and the general population.
- RQ-B: Compare fatigue, sleep, physical activity, and physical and mental HRQoL between treatment modalities within the CKD population.
- RQ-C: Investigate whether fatigue mediates the relationship between physical activity and physical and mental HRQoL.
- RQ-D: Investigate whether sleep moderates the relationship between physical activity and fatigue.
- RQ-E: If RQ-C and RQ-D are confirmed, examine whether sleep moderates the indirect relationship—mediated by fatigue—between physical activity and HRQoL.

## Materials and methods

### Study design

The study employed a cross-sectional design. Patients were recruited through the official German national patient association for kidney disease (Bundesverband Niere e.V.), in cooperation with Fresenius Medical Care Germany. An information letter, informed consent form, and the study questionnaire were published in the association’s quarterly journal *Der Nierenpatient*. Patients could detach the questionnaire from the journal, sign the informed consent form prior to completing it, and return both anonymously in a prepaid envelope to the patient association. Alternatively, after providing informed consent, participants had the option to complete the survey online via the survey tool LimeSurvey. Thus, participants could choose their preferred mode of participation and were given ten weeks to respond following publication in the journal. Inclusion criteria were age ≥18 years, a diagnosis of CKD, and residence in Germany. CKD status and treatment modality were self-reported.

A power analysis was conducted using GPower (32). Under a significant level of.05, a power of 95%, in total four covariates and medium effect size, a minimum of total 357 participants was required.

Ethical approval for the study was obtained from the Psychology Research Ethics Committee of Leiden University (proposal number CEP 18-0516/257). Data will be shared upon reasonable request to the first author.

### Measures

The Short Form (SF)-12 (33) was used to assess physical and mental HRQoL. Twelve statements with varying response categories were converted into physical and mental standardized values. From the standardized values, a norm-based T-score is calculated for the physical and mental component, with higher scores indicating a better HRQoL. The SF-12 demonstrated good reliability, construct validity, and responsiveness (34) and test-retest reliability was supported in clinical patients (35).

Physical activity was assessed with the International Physical Activity Questionnaire-Short Form (IPAQ-SF), a valid and reliable tool (36) providing information on the time spent on moderate and vigorous-intensive physical activity, walking and sitting.

Fatigue was measured using the 20-item Multidimensional Fatigue Inventory (MFI-20) (37), consisting of five scales: general fatigue, physical fatigue, mental fatigue, reduced activity, and reduced motivation. A total MFI score can also be computed. Items were scored on a five-point scale (1 = not true, …, 5 = true). Reversely stated items were recoded and a higher score indicates more fatigue. The instrument has adequate reliability and validity (38). In the current study, reliability is good for all subscales (*α* = 0.77–0.84) except for reduced motivation (*α* = 0.61), which has acceptable internal consistency reliability.

Sleep quality was assessed with the 4-item sleep scale of the Kidney Disease Quality of Life - Short Form (KDQOL-SF) (39). Participants rated their sleep quality on a 0 to 10 scale (0 = bad, …, 10 = excellent) and three statements on other sleep problems in the last four weeks using a 6-point scale (1 = never, …, 6 = always). Scores were transformed and summed on a 0 to 100 scale. In the current study, internal consistency reliability is good (*α* = 0.75). Quantity of sleep was measured by self-reported effective sleeping time in hours.

### Statistical analysis

Normative data of the healthy German population on physical and mental HRQoL (40), physical activity (41), fatigue (42) and sleep quantity (43), was compared with the sample data of the study (RQ-A). For the total sample and across gender groups, the average HRQoL score was tested against the population mean using one-sample T-tests. A one-sample Wilcoxon signed-rank test was used to evaluate physical activity against the reported median of the German population. Norm scores for fatigue were reported across age and gender, and were compared within each subgroup using two-samples T-tests. No norm-reference data was available on sleep quality. For the purpose of comparison, sleep quantity (effective sleeping time) of CKD patients was transformed into the categories “short”, “optimal” and “long” as Schlack and colleagues (43) only provided categorical reference data. This study’s sample distribution was tested against their reported percentages using a multinomial goodness-of-fit Chi-squared test. As measure of effect size, the Cohen’s d was used when applying T-tests (≥ 0.20 as “small”; ≥ 0.50 as “medium”; ≥ 0.8 as “large”); phi was used when a Chi-squared test was conducted (0.10 ~ small; 0.30 ~ medium; 0.50 ~ large). To control for multiple testing, *p*-values were adjusted using Bonferroni corrections.

For the multivariate analyses, a logarithmic transformation was applied to the physical activity variable in accordance with the recommendations of Tabachnick and Fidell (44). To compare fatigue, sleep, physical activity, and physical and mental HRQoL between treatment modalities, ANCOVAs were conducted. The PROCESS macro developed by Hayes (45) was used to test mediation (RQ-C), moderation, and moderated mediation (RQ-D and RQ-E). These analyses, were performed for each aspect of fatigue separately to avoid issues of collinearity among the different MFI (sub)scales. All multivariate analyses were controlled for gender, age, comorbidity, and disease duration. For each model, the assumptions of linearity, homoscedasticity, and normality of residuals were inspected using residual-versus-fitted plots and partial regression plots. Variance Inflation Factor values indicated no problems with multicollinearity (all VIFs < 2). Missing data were handled using pairwise deletion, so that all available data for each analysis could be included.

Statistical analyses were conducted using IBM SPSS Statistics version 22 with the PROCESS macro version 4.2 (45).

## Results

### Subjects

A total of 465 patients participated in the study. The average age was 53.78 years (*SD* = 15.00); 50.3 percent were women. Demographic and medical characteristics of the patient sample can be found in **Table 1**.

**Table 1:** Demographic and medical characteristics of the sample (N = 465).

Demographic characteristics		n	%	M	SD	Minimum	Maximum
Age				53.78	15.00	14	87
Gender	Male	229	49.7%				
	Female	232	50.3%				
Occupation	Student	27	5.9%				
	Employee	157	34.1%				
	Retiree	228	49.6%				
	Unemployed	10	2.2%				
	Other	38	8.3%				
*Medical background information*		*n*	%	*Median*	IQR	Minimum	Maximum
Disease duration (in years)				22.5	14	0	60
Comorbidities	None present	91	19.7%				
	Present	372	80.3%				
Treatment	Transplantation	112	26.2%				
	Hemodialysis	239	55.8%				
	Peritoneal dialysis	28	6.5%				
	Patients without RRT	49	11.4%				
	Unknown	37	8.0%				

RRT, Renal Replacement Therapy; IQR, Interquartile Range.

### Norm-comparisons of CKD patients with the German population

In the CKD patient group, the mean HRQoL scores were significantly lower than the normative means for the physical (*M* = 39.32 vs. 51.40) and mental component (*M* = 45.55 vs. 49.30). The associated effect size was medium to large for the physical (*d* = -1.13), and small for the mental component (*d* = -0.33). The same pattern of differences was found within the female and male subsamples, respectively (all *p*s <.001).

Physical activity was significantly lower in the patient group than in the general population. The median score in the patient sample was 2091 (IQR = 3412), which is significantly lower than the median of 5070 reported by Rütten et al. (41) (*p* <.001). The CKD sample mean (*M* = 3249.7, *SD* = 3925.1) was also substantially lower than the reported population mean (*M* = 8534.2, *SD* = 9024.5).

**Figure 1:**
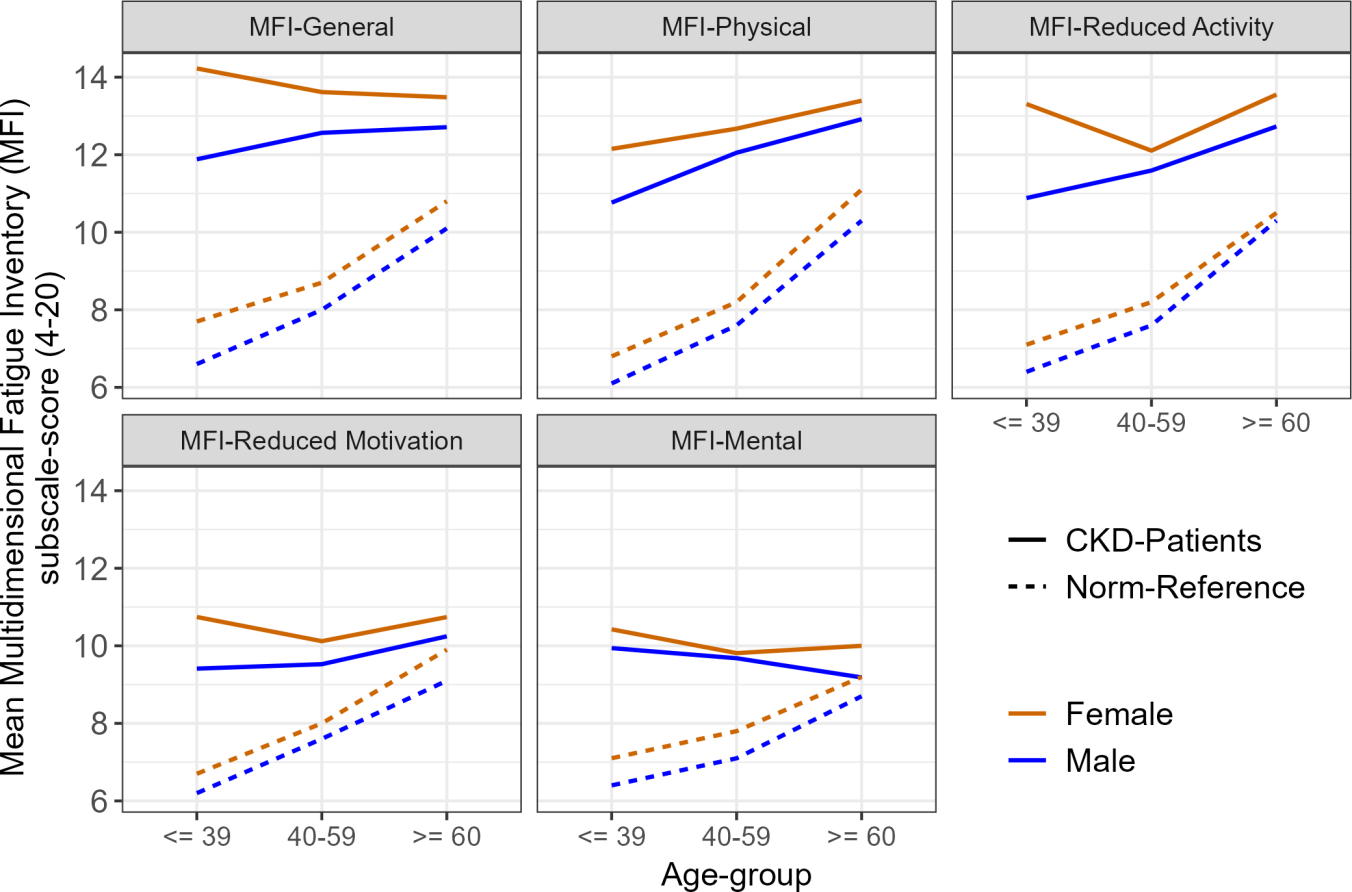
Means of MFI-subscale scores in the CKD sample compared to norm-reference values within age and gender groups.

An overview of norm comparisons of the MFI subscales, stratified by gender and age, is presented in **Figure 1**. Higher fatigue was reported in the sample of CKD patients, except for reduced motivation and mental fatigue for the oldest group of women and mental fatigue for the oldest group of men. With respect to the age-groups, the largest differences were found in younger men and women (≤ 40 years; Cohen’s *d* = 1.02–1.97). With respect to gender, a similar pattern was found for females and males. With respect to the MFI-subscales, overall, the largest differences were found for general fatigue (*d* = 0.71–1.97), followed by reduced activity (*d* = 0.62–1.97) and physical fatigue (*d* = 0.55–1.68); the differences for reduced motivation (*d* = 0.24–1.44) and mental fatigue (*d* = 0.13–1.21) were smaller.

A significant difference in sleeping time was found between the CKD sample and the healthy German population, χ²(2) = 32.52, p <.001; φ = 0.27. Short sleep (≤ 5 hours) was more common in the CKD group (21.1% vs. 12.3%), while optimal sleep was less frequent (72.2% vs. 81.6%). Long sleep (≥ 9 hours) occurred at similar rates (6.2% vs. 6.1%). This pattern was significant for both females, χ²(2) = 36.36, p <.001, φ = 0.40, and males, χ²(2) = 6.05, p = .049, φ = 0.17, with a larger effect for females. Female patients reported optimal sleep less often (68.4%) than male patients (76.6%) compared to the norm (80.5%).

### Comparisons across modalities of treatment

**Table 2** shows results of mean comparison tests across treatment modalities. Overall, transplant patients had the highest sleep quality, the lowest levels of (general) fatigue and reduced motivation, and the highest mental HRQoL, followed by hemodialysis patients. In contrast, the lowest sleep quality and mental HRQoL, and the highest (general) fatigue was found in the group of patients without RRT. Level of physical activity, physical fatigue, reduced activity, mental fatigue and the physical component of HRQoL was not significantly different across treatment modalities.

**Table 2:** Differences between treatment modalities for physical activity, sleep, fatigue and health-related quality of life.

Variable	Procedure								Mean comparison		
	Transplantation		Hemo-dialysis		Peritoneal dialysis		Patients without RRT		ANCOVA test result		
	*M*	*SD*	*M*	*SD*	*M*	*SD*	*M*	*SD*	*F*-value	*p*	*Eta* ^2^
Physical Activity	7.56	1.18	7.49	1.22	7.66	0.93	7.20	1.34	0.40	0.75	0.004
Sleep quality	63.24	19.20	57.56	19.03	53.70	23.12	51.41	19.50	2.93*	0.03	0.023
MFI-G	12.17	4.29	13.18	3.79	13.85	3.66	14.63	3.39	4.01**	0.008	0.032
MFI-Physical	11.60	4.55	12.69	4.04	12.89	3.21	13.30	3.82	2.51	0.06	0.020
MFI-RA	11.50	4.42	12.73	3.87	12.46	3.36	12.96	3.94	2.23	0.08	0.018
MFI-RM	9.22	3.70	10.39	3.56	10.70	2.45	10.40	2.99	3.78*	0.01	0.030
MFI-M	9.23	4.12	9.70	3.93	9.86	3.92	10.37	3.57	1.38	0.25	0.011
MFI-tot	53.68	18.51	58.67	16.03	59.78	13.49	61.56	14.32	3.54*	0.02	0.030
SF-12 Physical	41.07	10.52	38.35	10.49	40.41	10.00	39.57	11.72	1.45	0.23	0.012
SF-12 Mental	48.06	11.51	45.23	11.13	46.48	11.74	43.30	10.44	2.87*	0.04	0.023

**p <.01, *p <.05; ANCOVA, Analysis of Covariance; MFI, Multidimensional Fatigue Inventory; RRT, Renal Replacement Therapy; MFI subscales: MFI-G, General; MFI-P, Physical; MFI-RA, Reduced Activity; MFI-RM, Reduced Motivation; MFI-M, Mental; MFI-tot, Total; SF-12 Physical, Physical Health-Related Quality of Life; SF-12 Mental, Mental Health-Related Quality of Life; Eta^2^, measure of effect size. ANCOVAs were controlled for effect of age, gender, comorbidity and disease duration.

### Fatigue as a mediator of the effect of physical activity on HRQoL

**Figure 2:**
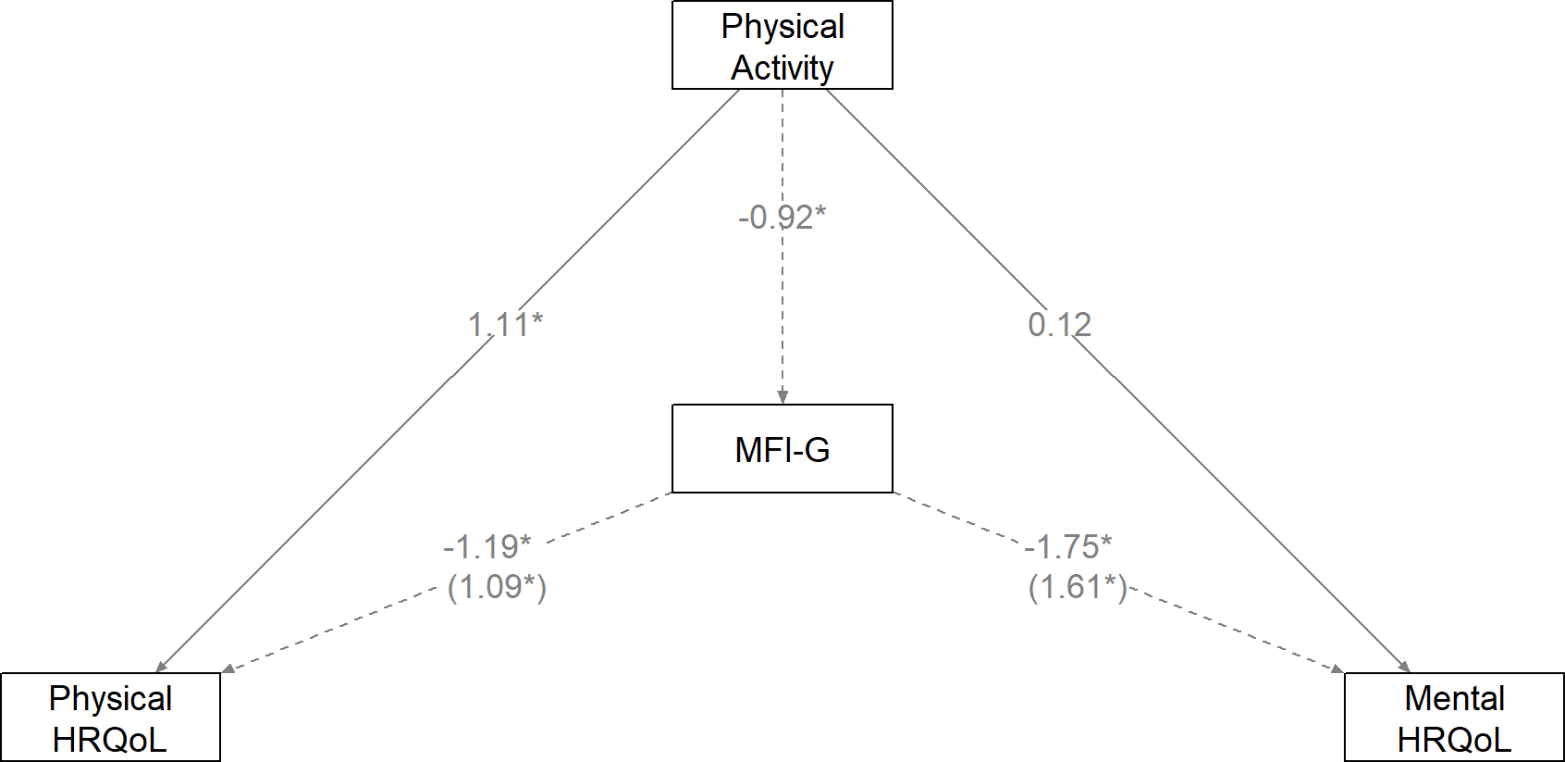
Unstandardized path coefficients of the mediating effect of fatigue (MFI-total) on the effect of physical activity on (the physical and mental component of) Health-Related Quality of Life (HRQoL). The indirect effects are shown in parenthesis (*p <.05).

In **Table 3** (Panel A), the results of the mediation analysis are presented. The indirect effects were all significantly positive. Patients who were physically more active, had a lower level of fatigue, which in turn was related to better physical and mental HRQoL. For physical quality of life, physical fatigue showed the strongest indirect association (*β* = 0.195); for the mental component of HRQoL, total fatigue showed the largest indirect association (*β* = 0.243). The smallest indirect effects were found for reduced motivation and mental fatigue. To illustrate the mediating effect of fatigue, results for general fatigue (MFI-G) are presented in **Figure 2**.

**Table 3:** Indirect effects of physical activity on HRQoL for each of the fatigue scales as mediator.

Panel A: mediation	Physical component score				Mental component score				
MFI scale	Effect^A^	SE_Boot_	95% CI	β	Effect^A^	SE_Boot_		95% CI	β
*General*	1.09*	0.19	(0.74; 1.50)	0.133	1.61*	0.27		(1.09; 2.15)	0.180
*Physical*	1.63*	0.25	(1.17; 2.15)	0.195	1.87*	0.28		(1.37; 2.45)	0.206
*Reduced Activity*	1.16*	0.23	(0.73; 1.65)	0.140	1.40*	0.28		(0.90; 2.00)	0.157
*Reduced Motivation*	0.45*	0.14	(0.20; 0.73)	0.054	1.22*	0.27		(0.69; 1.76)	0.135
*Mental*	0.30*	0.12	(0.10; 0.57)	0.036	1.07*	0.32		(0.46; 1.71)	0.119
*Total*	1.29*	0.22	(0.86; 1.76)	0.153	2.23*	0.33		(1.58; 2.88)	0.243
Panel B: moderation									
Moderator	Sleep Quality				Sleep Quantity				
MFI scale	*F*-value	*p*	*R^2^* change		*F*-value		*p*	*R^2^* change	
*General*	1.73	0.19	0.004		0.73		0.40	0.002	
*Physical*	5.38*	0.02	0.011		1.44		0.23	0.004	
*Reduced Activity*	4.92*	0.03	0.012		2.18		0.14	0.006	
*Reduced Motivation*	2.39	0.12	0.006		1.81		0.18	0.005	
*Mental*	0.09	0.76	<0.001		0.63		0.43	0.002	
*Total*	2.22	0.14	0.005		0.62		0.43	0.002	
Panel C: moderated mediation (sleep quality)									
	Physical Component Score				Mental Component Score				
MFI scale	Effect^B^	SE_Boot_	95% CI		Effect^B^		SE_Boot_	95% CI	
*General*	0.012	0.009	(-0.006; 0.030)		0.017		0.013	(-0.008; 0.043)	
*Physical*	0.024*	0.010	(0.007; 0.046)		0.026*		0.011	(0.007; 0.050)	
*Reduced Activity*	0.019*	0.009	(0.004; 0.038)		0.022*		0.010	(0.004; 0.043)	
*Reduced Motivation*	0.008	0.005	(-0.002; 0.019)		0.020		0.013	(-0.005; 0.048)	
*Mental*	-0.001	0.005	(-0.011; 0.008)		-0.005		0.015	(-0.032; 0.027)	
*Total*	0.014	0.009	(-0.002; 0.033)		0.023		0.014	(-0.003; 0.054)	

*p <.05; β, standardized indirect effect; CI, Confidence Interval; SE_Boot_, bootstrapped standard error; MFI, Multidimensional Fatigue Inventory; HRQoL, Health-Related Quality of Life. ^A^ indirect effect of Physical Activity on Quality of Life through the fatigue; ^B^ index of moderated mediation by sleep quality. All models controlled for Comorbidity, Gender, Age and Disease Duration.

### Sleep as a moderator of the effect of physical activity on fatigue

**Figure 3:**
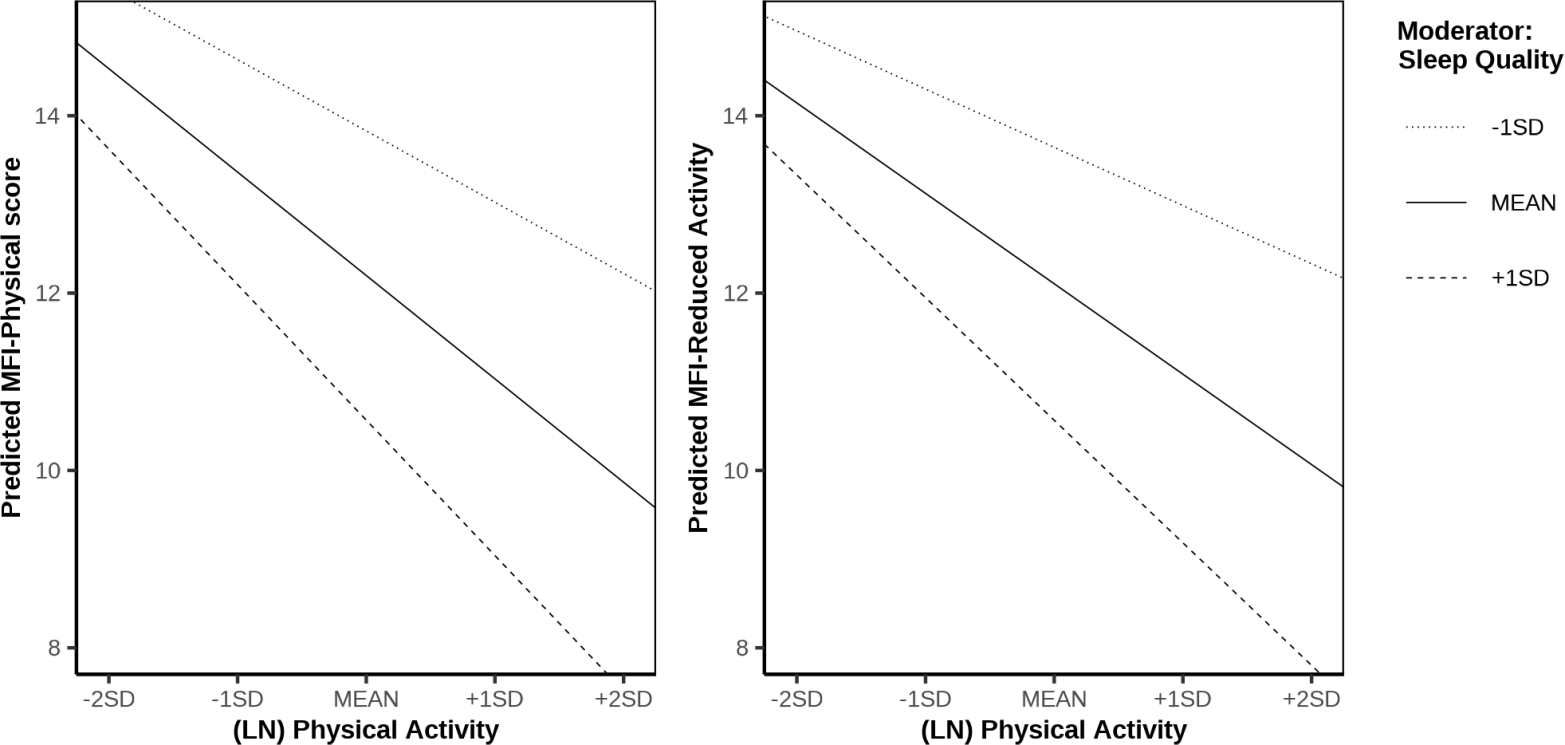
Interaction plots of physical activity × sleep quality on the MFI-physical subscale (left) and physical activity × sleep quality on the MFI-reduced activity subscale score (right).

Panel B (**Table 3**) gives an overview of the moderating effects of sleep quality and sleep quantity. For patients reporting better sleep quality, the association between physical activity and physical fatigue was stronger, indicating a moderating effect of sleep quality (see **Figure 3**, left). A similar moderating effect was observed for the MFI subscale measuring reduced activity: patients with better sleep quality and higher physical activity levels showed more favorable scores on this subscale (see **Figure 3**, right). By contrast, sleep quantity did not significantly moderate the association between physical activity and fatigue.

### Sleep quality as a moderator of the indirect effect of physical activity on physical and mental HRQoL via fatigue

The results of the analysis are shown in **Table 3** (Panel C). The indirect association between physical activity and physical and mental HRQoL via physical fatigue was moderated by sleep quality. Specifically, for patients with better sleep quality, the negative association between physical activity and physical fatigue was stronger, which in turn was related to higher HRQoL. A similar moderated mediation was observed for the MFI subscale measuring reduced activity: patients with higher sleep quality and greater physical activity reported less reduced activity, which was associated with higher physical and mental HRQoL.

## Discussion

The results of the current study indicate that CKD patients report lower mental and physical HRQoL compared to a sample from the general population, with the largest difference observed for physical HRQoL. CKD patients also report lower levels of physical activity and higher levels of fatigue. The greatest differences in fatigue were found among patients younger than 40 years. In addition, a larger proportion of participants in our sample reported short sleep duration (less than five hours per night) compared to data from the general population.

A comparison between the different treatment modalities shows that transplant patients report the best sleep quality, the least fatigue (with respect to general fatigue and reduced motivation) and the best mental HRQoL, followed by the hemodialysis patients. Patients without RRT are doing worse on each of these dimensions. No differences were found for degree of physical activity, the physical component of quality of life, and the other dimensions of fatigue.

Results further indicate that higher physical activity is linked to lower fatigue, which in turn is associated with better mental and physical HRQoL. For the mental component, the strongest indirect association was with total fatigue, whereas for the physical component it was with physical fatigue. The association between higher physical activity and lower fatigue (physical fatigue and reduced activity), which in turn is related to higher HRQoL, appeared more pronounced in patients reporting better sleep quality, with somewhat stronger effects for mental than for physical HRQoL.

The finding that CKD patients experience more fatigue than the general population is consistent with the existing literature on the subject (2, 3). However, the present study shows that the largest differences between CKD patients and the general population occur in a younger age group, an effect that, to our knowledge, has not been described before. A possible explanation for our finding is that younger patients may experience a heavier impact of the disease on their functioning and, as a consequence, hold more negative thoughts about their condition and related symptoms, which in turn may cause them to experience more severe fatigue. The latter is consistent with the biopsychosocial model of fatigue in CKD patients, which implies that psychological factors, including cognitions, play an important role in the perpetuation of fatigue (2, 5).

The comparison of the different treatment modalities shows that transplant patients do better whereas patients without RRT do worse than the other subgroups in a number of respects. The finding regarding transplant patients is consistent with previous studies (17, 46). That patients not yet on RRT do worse may seem counterintuitive. It can however be explained by the fact that these patients already experience symptoms due to deteriorating kidney function but are not yet receiving treatment that can adequately alleviate these symptoms (47). The null findings for degree of physical activity, the physical component of quality of life, and the other dimensions of fatigue may indicate that such differences truly do not exist between treatment modalities, or they may reflect limitations of the study. In particular, some subgroups (e.g., patients on peritoneal dialysis or not yet on RRT) were relatively small, which reduces statistical power and sensitivity to detect potential differences. It is therefore possible that the study was underpowered to capture more subtle contrasts. Nevertheless, future research with larger and more balanced samples across treatment modalities will be important to clarify whether such group differences are truly absent or simply not detected in the current study.

Previous research has reported positive associations between physical activity and both fatigue (9) and quality of life in CKD patients (21). In addition, fatigue in CKD patients has been shown to be associated with lower quality of life (18). However, to our knowledge, this is the first study to examine these relationships together and to suggest that fatigue may serve as a pathway linking physical activity with quality of life, that is, the association between physical activity and HRQoL may be explained, at least in part, by fatigue.

Although earlier studies have shown that poorer sleep quality is associated with higher patient-reported fatigue (13, 28, 29), as well as lower quality of life (23) in CKD patients, the question of whether sleep quality modifies the relationship between physical activity, fatigue, and HRQoL has not previously been examined. The present study’s finding that the strength of the association between physical activity and fatigue (and thereby HRQoL) appears to vary depending on sleep quality is therefore noteworthy and aligns with findings in non-clinical populations (30, 31).

### Strengths and weaknesses

A major strength of the study is that physical activity, fatigue, and sleep, factors that are all known to influence HRQoL as well as disease progression and mortality in CKD patients (18, 20, 27), were examined together. Another strength is the inclusion of a heterogeneous CKD population, consisting of patients on hemodialysis, peritoneal dialysis, and kidney transplantation, as well as patients not yet receiving RRT. This contrasts with most prior studies, which have typically focused only on hemodialysis populations.

At the same time, several limitations should be acknowledged. First, while mediation and moderation analyses provide valuable insights into potential pathways, the cross-sectional design of the study does not allow conclusions regarding temporal sequence or causality. Longitudinal studies are needed to establish causal inferences. Second, recruitment took place via a patient association newsletter and an anonymous online survey, which may have introduced self-selection bias, for example, by attracting more motivated or health-literate patients. This, in turn, may limit the generalizability of our findings. Third, physical activity was measured using the IPAQ-SF, a widely used tool, but one known to overestimate activity and subject to recall bias. Similarly, sleep was assessed with the abbreviated 4-item KDQOL sleep scale, which may not fully capture all dimensions of sleep quality. Fourth, all measures in the study - including CKD status and treatment modality - were self-reported; no objective measures such as actigraphy for activity (using an accelerometer) or polysomnography for sleep were employed. Additional medical information such as CKD stage and medication use was not available, nor were bloodwork and clinical data (e.g., hemoglobin, eGFR), as the study was conducted anonymously in collaboration with a patient organization. This limits the ability to account for important medical confounders; for instance, anemia or uremic toxins may contribute to fatigue independently of physical activity or sleep. Finally, some CKD subgroups were relatively small, which may have reduced the sensitivity to detect subtle differences across treatment modalities.

### Suggestions for future research and potential clinical implications

Beyond statistical significance, the present findings also have clinical relevance. Fatigue and sleep quality emerged as important factors associated with physical activity and HRQoL in patients with CKD. Although some of the observed effect sizes were modest, they highlight domains directly relevant to patients’ daily functioning and well-being. For example, even small improvements in sleep quality may strengthen the association between physical activity and reduced fatigue, which can translate into better energy levels, adherence to treatment, and overall quality of life. Building on this, the results suggest that interventions incorporating physical activity could be a promising strategy to address both fatigue and quality of life in CKD patients.

At the same time, prescribing exercise to fatigued patients with poor sleep quality may be challenging in practice. Tailored approaches that account for sleep quality, and that integrate sleep-focused strategies before or alongside physical activity, may therefore be more feasible. While previous studies have established that fatigue is related to lower QoL and that exercise is associated with reduced fatigue in CKD (24), the present study extends this work by examining mediated and moderated associations. These findings underscore the relevance of fatigue as a potential pathway and sleep quality as a potential modifier in the relationship between physical activity and HRQoL. To investigate these relationships more rigorously, it will be important to conduct longitudinal studies to further examine the observed associations and to establish temporal sequence and causality. In addition, the use of objective measures for physical activity and sleep would strengthen the validity of future findings. Subsequently, intervention studies are needed to test whether interventions targeting sleep and physical activity have the desired effect on reducing fatigue.

The results also show a substantial gap in fatigue between younger CKD patients and their peers in the general population, suggesting that younger patients may be a primary target group for intervention development. Moreover, the impact of untreated fatigue on later treatment outcomes has not yet been adequately studied from earlier stages of CKD through to renal replacement therapy. Addressing this gap would allow for the more targeted development of interventions across the disease trajectory.

For future research, it will be important to include biological indicators of renal function in addition to the psychosocial variables examined here. This would make it possible to investigate a broader range of factors within the biopsychosocial model of fatigue in CKD patients.

## Data Availability

The datasets presented in this article are not readily available because they contain potentially identifiable participant information. Requests to access the datasets should be directed to the corresponding author.

## References

[1] Finsterer J Mahjoub SZ . Fatigue in healthy and diseased individuals. Am J Hospice Palliative Med. (2014) 31:562–75. doi: 10.1177/1049909113494748, PMID: 23892338

[2] Artom M Moss-Morris R Caskey F Chilcot J . Fatigue in advanced kidney disease. Kidney Int. (2014) 86:497–505. doi: 10.1038/ki.2014.86, PMID: 24694985

[3] Joshwa B Campbell ML . Fatigue in patients with chronic kidney disease: evidence and measures. Nephrol Nurs J. (2017) 44:337–44., PMID: 29160968

[4] Ju A Unruh M Davison S Dapueto J Dew MA Fluck R . Establishing a core outcome measure for fatigue in patients on hemodialysis: A standardized outcomes in nephrology–hemodialysis (SONG-HD) consensus workshop report. Am J Kidney Dis. (2018) 72:104–12. doi: 10.1053/j.ajkd.2017.12.018, PMID: 29551585

[5] Picariello F Moss-Morris R Macdougall IC Chilcot J . The role of psychological factors in fatigue among end-stage kidney disease patients: A critical review. Clin Kidney J. (2017) 10:79–88. doi: 10.1093/ckj/sfw113, PMID: 28638608 PMC5469558

[6] Romagnani P Remuzzi G Glassock R Levin A Jager KJ Tonelli M . Chronic kidney disease. Nat Rev Dis Primers. (2017) 3:e17088. doi: 10.1038/nrdp.2017.88, PMID: 29168475

[7] Davey CH Webel AR Sehgal AR Voss JG Huml A . Fatigue in individuals with end stage renal disease. Nephrol Nurs J: J Am Nephrol Nurses’ Assoc. (2019) 46:497–508. PMC7047987 31566345

[8] Bossola M Arena M Urciuolo F Antocicco M Pepe G Calabrò GE . Fatigue in kidney transplantation: A systematic review and meta-analysis. Diagnostics. (2021) 11(5):833. doi: 10.3390/diagnostics11050833, PMID: 34063041 PMC8147914

[9] Gregg LP Bossola M Ostrosky-Frid M Hedayati SS . Fatigue in CKD. Clin J Am Soc Nephrol. (2021) 16:1445–55. doi: 10.2215/CJN.19891220, PMID: 33858827 PMC8729574

[10] Bonner A Wellard S Caltabiano M . The impact of fatigue on daily activity in people with chronic kidney disease. J Clin Nurs. (2010) 19:3006–15. doi: 10.1111/j.1365-2702.2010.03381.x, PMID: 21040007

[11] Gregg LP Jain N Carmody T Minhajuddin AT Rush AJ Trivedi MH . Fatigue in nondialysis chronic kidney disease: correlates and association with kidney outcomes. Am J Nephrol. (2019) 50:37–47. doi: 10.1159/000500668, PMID: 31167183 PMC6620124

[12] Bonner A Caltabiano M Berlund L . Quality of life, fatigue, and activity in Australians with chronic kidney disease: A longitudinal study. Nurs Health Sci. (2013) 15:360–7. doi: 10.1111/nhs.12038, PMID: 23480135

[13] Jhamb M Pike F Ramer S Argyropoulos C Steel J Dew MA . Impact of fatigue on outcomes in the hemodialysis (HEMO) study. Am J Nephrol. (2011) 33:515–23. doi: 10.1159/000328004, PMID: 21555875 PMC4484241

[14] Brys ADH Lenaert B Van Heugten CM Gambaro G Bossola M . Exploring the diurnal course of fatigue in patients on hemodialysis treatment and its relation with depressive symptoms and classical conditioning. J Pain Symptom Manage. (2019) 57:890–8. doi: 10.1016/j.jpainsymman.2019.02.010, PMID: 30776536

[15] Picariello F Norton S Moss-Morris R Macdougall IC Chilcot J . Fatigue in prevalent haemodialysis patients predicts all-cause mortality and kidney transplantation. Ann Behav Med. (2019) 53:501–14. doi: 10.1093/abm/kay061, PMID: 30020399

[16] SChade van Westrum E Hoogeveen EK Broekman BFP Siegert CEH Keurhorst D Annema C . Fatigue across different chronic kidney disease populations: experiences and needs of patients. Clin Kidney J. (2025) 18:sfaf118. doi: 10.1093/ckj/sfaf118, PMID: 40599823 PMC12209799

[17] Maglakelidze N Pantsulaia T Tchokhonelidze I Managadze L Chkhotua A . Assessment of health-related quality of life in renal transplant recipients and dialysis patients. Transplant Proc. (2011) 43:376–9. doi: 10.1016/J.TRANSPROCEED.2010.12.015, PMID: 21335226

[18] Kraus MA Fluck RJ Weinhandl ED Kansal S Copland M Komenda P . Intensive hemodialysis and health-related quality of life. Am J Kidney Dis. (2016) 68:33–42. doi: 10.1053/j.ajkd.2016.05.023, PMID: 27772641

[19] Kosmadakis GC Bevington A Smith AC Clapp EL Viana JL Bishop NC . Physical exercise in patients with severe kidney disease. Nephron - Clin Pract. (2010) 115:7–16. doi: 10.1159/000286344, PMID: 20173344

[20] Zelle DM Klaassen G Van Adrichem E Bakker SJL Corpeleijn E Navis G . Physical Inactivity: A risk factor and Target for Intervention in Renal Care. Nat Rev Nephrol. (2017) 13:152–68. doi: 10.1038/nrneph.2016.187, PMID: 28138130

[21] Barcellos FC Santos IS Umpierre D Bohlke M Hallal PC . Effects of exercise in the whole spectrum of chronic kidney disease: a systematic review. Clin Kidney J. (2015) 8:753–65. doi: 10.1093/ckj/sfv099, PMID: 26613036 PMC4655802

[22] Bündchen DC Sousa H Afreixo V Frontini R Ribeiro O Figueiredo D . Intradialytic exercise in end-stage renal disease: an umbrella review of systematic reviews and/or meta-analytical studies. Clin Rehabil. (2021) 35:812–28. doi: 10.1177/0269215520986784, PMID: 33530715

[23] Yamamoto R Ito T Nagasawa Y Matsui K Egawa M Nanami M . Efficacy of aerobic exercise on the cardiometabolic and renal outcomes in patients with chronic kidney disease: A systematic review of randomized controlled trials. J Nephrol. (2021) 34:155–64. doi: 10.1007/s40620-020-00865-3, PMID: 33387341

[24] Zhao Q-G Zhang H-R Wen X Wang Y Chen X-M Chen N . Exercise interventions on patients with end-stage renal disease: a systematic review. Clin Rehabil. (2019) 33:147–56. doi: 10.1177/0269215518817083, PMID: 30789077

[25] Roumelioti M-E Argyropoulos CP Unruh ML . Sleep disorders in patients with CKD and ESRD. In: Cukor D Cohen SD Kimmel PL , eds. Psychosocial Aspects of Chronic Kidney Disease. Cambridge, MA: Academic Press (Elsevier) (2021). p. 183–212. doi: 10.1016/B978-0-12-817080-9.00009-9

[26] Elder SJ Pisoni RL Akizawa T Fissell R Andreucci VE Fukuhara S . Sleep quality predicts quality of life and mortality risk in haemodialysis patients: results from the dialysis outcomes and practice patterns study (DOPPS). Nephrol Dialysis Transplant. (2008) 23:998–1004. doi: 10.1093/ndt/gfm630, PMID: 17911092

[27] Yamamoto R Shinzawa M Isaka Y Yamakoshi E Imai E Ohashi Y . Sleep quality and sleep duration with CKD are associated with progression to ESKD. Clin J Am Soc Nephrol. (2018) 13:1825–32. doi: 10.2215/CJN.01340118, PMID: 30442866 PMC6302324

[28] Jhamb M Liang K Yabes J Steel JL Dew MA Shah N . Prevalence and correlates of fatigue in chronic kidney disease and end-stage renal disease: are sleep disorders a key to understanding fatigue? Am J Nephrol. (2013) 38:489–95. doi: 10.1159/000356939, PMID: 24335380 PMC3925636

[29] Brys ADH Stifft F Van Heugten CM Bossola M Gambaro G Lenaert B . mHealth-based experience sampling method to identify fatigue in the context of daily life in haemodialysis patients. Clin Kidney J. (2021) 14:245–54. doi: 10.1093/ckj/sfaa124, PMID: 33564425 PMC7857808

[30] Miller M Lee-Chambers J Cooper B Boolani A Jansen E . Associations between physical activity and energy and fatigue depend on sleep quality. Fatigue: Biomed Health Behav. (2020) 8:193–204. doi: 10.1080/21641846.2020.1843790

[31] Herring MP Monroe DC Kline CE O’Connor PJ MacDonncha C . Sleep quality moderates the association between physical activity frequency and feelings of energy and fatigue in adolescents. Eur Child Adolesc Psychiatry. (2018) 27:1425–32. doi: 10.1007/s00787-018-1134-z, PMID: 29508054 PMC6410735

[32] Faul F Erdfelder E Buchner A Lang AG . Statistical power analyses using G* Power 3.1: Tests for correlation and regression analyses. Behav Res Methods. (2009) 41:1149–60. doi: 10.3758/BRM.41.4.1149, PMID: 19897823

[33] Ware JE Kosinski M Keller SD . A 12-item short-form health survey: construction of scales and preliminary tests of reliability and validity. Med Care. (1996) 34:220–33. doi: 10.1097/00005650-199603000-00003, PMID: 8628042

[34] Luo X George ML Kakouras I Edwards CL Pietrobon R Richardson W . Reliability, validity, and responsiveness of the short form 12-item survey (SF-12) in patients with back pain. Spine. (2003) 28:1739–45. doi: 10.1097/01.BRS.0000083169.58671.96, PMID: 12897502

[35] Bohannon RW Maljanian R Landes M . Test-retest reliability of short form (SF)-12 component scores of patients with stroke. Int J Rehabil Res. (2004) 27:149–50. doi: 10.1097/01.MRR.0000127350.25287.08, PMID: 15167113

[36] Sember V Meh K Sorić M Jurak G Starc G Rocha P . Validity and reliability of international physical activity questionnaires for adults across EU countries: systematic review and meta analysis. Int J Environ Res Public Health. (2020) 17:7161. doi: 10.3390/IJERPH17197161, PMID: 33007880 PMC7579664

[37] Smets EMA Garssen B Bonke B De Haes JCJM . The multidimensional fatigue inventory (MFI) psychometric qualities of an instrument to assess fatigue. J Psychosom Res. (1995) 39:315–25. doi: 10.1016/0022-3999(94)00125-O, PMID: 7636775

[38] Hinz A Benzing C Brähler E Zenger M Herzberg PY Finck C . Psychometric properties of the multidimensional fatigue inventory (MFI-20), derived from seven samples. J Pain Symptom Manage. (2020) 59:717–23. doi: 10.1016/J.JPAINSYMMAN.2019.12.005, PMID: 31837450

[39] Hays RD Kallich JD Mapes DL Coons SJ Carter WB . Development of the kidney disease quality of life (KDQOL) instrument. Qual Life Res: Int J Qual Life Aspects Treatment Care Rehabil. (1994) 3:329–38. doi: 10.1007/BF00451725, PMID: 7841967

[40] Ellert U Kurth BM . Gesundheitsbezogene lebensqualität bei erwachsenen in Deutschland. Bundesgesundheitsblatt - Gesundheitsforschung - Gesundheitsschutz. (2013) 56:643–9. doi: 10.1007/s00103-013-1700-y, PMID: 23703481

[41] Rütten A Ziemainz H Schena F Stahl T Stiggelbout M Auweele Y . Using different physical activity measurements in eight european countries. Results of the European physical activity surveillance system (EUPASS) time series survey. Public Health Nutr. (2003) 6:371–6. doi: 10.1079/phn2002450, PMID: 12795825

[42] Schwarz R Krauss O Hinz A . Fatigue in the general population. Onkologie. (2003) 26:140–4. doi: 10.1159/000069834, PMID: 12771522

[43] Schlack R Hapke U Maske U Busch M Cohrs S . Häufigkeit und Verteilung von Schlafproblemen und Insomnie in der deutschen Erwachsenenbevölkerung. Bundesgesundheitsblatt - Gesundheitsforschung - Gesundheitsschutz. (2013) 56:740–8. doi: 10.1007/s00103-013-1689-2, PMID: 23703493

[44] Tabachnick BG Fidell LS . Using multivariate statistics. 7th ed. New York, NY: Pearson (2019).

[45] Hayes AF . An index and test of linear moderated mediation. Multivariate Behav Res. (2015) 50:1–22. doi: 10.1080/00273171.2014.962683, PMID: 26609740

[46] Fujisawa M Ichikawa Y Yoshiya K Isotani S Higuchi A Nagano S . Assessment of health-related quality of life in renal transplant and hemodialysis patients using the SF-36 health survey. Urology. (2000) 56:201–6. doi: 10.1016/S0090-4295(00)00623-3, PMID: 10925078

[47] Grams ME Surapaneni A Appel LJ Lash JP Hsu J Diamantidis CJ . Clinical events and patient-reported outcome measures during CKD progression: findings from the chronic renal insufficiency cohort study. Nephrol Dialysis Transplant. (2021) 36:1685–93. doi: 10.1093/NDT/GFAA364, PMID: 33326030 PMC8396398

